# Functional mitral regurgitation: predictor for atrial substrate remodeling and poor ablation outcome in paroxysmal atrial fibrillation

**DOI:** 10.1097/MD.0000000000004333

**Published:** 2016-07-29

**Authors:** Yu Qiao, Lingmin Wu, Bingbo Hou, Wei Sun, Lihui Zheng, Ligang Ding, Gang Chen, Shu Zhang, Yan Yao

**Affiliations:** State Key Laboratory of Cardiovascular Disease, Cardiac Arrhythmia Center, Fuwai Hospital, National Center for Cardiovascular Diseases, Chinese Academy of Medical Sciences and Peking Union Medical College, Beijing, People's Republic of China.

**Keywords:** atrial fibrillation, catheter ablation, functional mitral regurgitation, voltage mapping

## Abstract

Functional mitral regurgitation (FMR) is not uncommon in atrial fibrillation (AF) patients. We sought to investigate the association between FMR and atrial substrate remodeling as well as the ablation outcome in paroxysmal AF (PAF) patients.

We retrospectively analyzed a prospectively enrolled cohort of 132 patients (age 55.1 ± 9.6 years, 75.8% male) with symptomatic PAF who underwent initial ablation in our institute. Functional mitral regurgitation was defined as regurgitation jet area to left atrium (LA) area ratio ≥ 0.1 without any primary valvular disease. Voltage mapping of LA was performed under sinus rhythm. Low voltage zones (LVZs) were semi-quantitatively estimated and presented as low voltage index. Follow-up for AF recurrence ≥ 12 months was performed.

In total, 40 patients (29.6%) were detected with FMR, who were older than the non-FMR patients (*P* = 0.007) and had larger LA diameters (*P* = 0.02). Left atrium LVZs were observed in 64.9% of patients with FMR versus 22.1% patients without FMR (*P* < 0.001). Functional mitral regurgitation independently predicted the presence of LVZs (OR 7.286; 95% CI 3.023–17.562; *P* < 0.001). During a mean follow-up of 22.9 ± 6.5 months, 38 patients (28.8%) experienced AF recurrence. The recurrence rate was 60.0% and 19.5% in FMR and non-FMR cohort, respectively (log rank *P* < 0.001). Multivariate analysis showed that FMR was an independent predictor for AF recurrence (HR 2.291; 95% CI 1.062–4.942; *P* = 0.03).

Functional mitral regurgitation was strongly associated with atrial substrate remodeling. Furthermore, patients with FMR have substantial risk for AF recurrence post ablation.

## Abstract 2

Supplemental Digital Content is available in the text

## 1 Introduction

Functional mitral regurgitation (FMR) is not an uncommon finding in patients with atrial fibrillation (AF).^[1–3]^ In the absence of primary valvular disease and left ventricular dysfunction, significant FMR could be identified in 7.4% to 29% of AF patients.^[1,2]^ Recent studies have shown that left atrial (LA) substrate remodeling and corresponding mitral valve annulus (MVA) dilation is the most possible cause of FMR, although this is still controversial.^[2,4]^ The fundamental work by Gertz et al^[1]^ revealed that significant FMR could be improved by sinus restoration, which rendered catheter ablation plausible for patients with FMR. However, previous studies demonstrated that patients with mitral regurgitation (MR) were more likely to experience recurrent AF post ablation, especially those with significant MR.^[5,6]^ Notably, these outcome studies included patients with both FMR and primary mitral abnormality-related MR. Whether FMR in patients without prior valvular impairment is associated with higher recurrence rate remains unclear.

The three-dimensional electro-anatomical mapping (EAM) is a surging technique for reflecting on substrate remodeling. Previous studies employing EAM and delayed enhancement-magnetic resonance imaging (DE-MRI) have shown that low voltage zones (LVZs) could serve as a surrogate for atrial remodeling and were associated with poor ablation outcome.^[7,8]^ Therefore, we sought to investigate the association between FMR and LVZs identified in EAM and the impact of isolated FMR on ablation outcome after circumferential pulmonary vein isolation (CPVI) procedure in patients with paroxysmal AF (PAF).

## 2 Methods

### 2.1 Study population

We conducted a retrospective analysis in a prospectively enrolled cohort, which contained 132 consecutive patients with symptomatic PAF who were presented in sinus rhythm and underwent the initial CPVI procedure in our center between October 2012 and March 2014. Previously ablated patients, patients comorbid with congenital heart disease or cardiomyopathy were excluded from the study. All patients gave written informed consent before the procedures. The study was approved by the institutional review board.

### 2.2 Echocardiography

A standard two-dimensional and Doppler echocardiography with color flow mapping was performed in every patient under sinus rhythm and patients who were presented with AF were excluded from study. Mitral regurgitation was evaluated under the recommendation of the American Society of Echocardiography's standards.^[9]^ Color Doppler scale and therefore Nyquist limit were determined by the clinical echocardiographer and in general were set to 50 to 70 cm/s.^[1]^ The ratio of maximum MR color jet area to LA area (MR/LA ratio) was calculated and classification of MR was defined as mild MR (MR/LA ratio ≥ 0.1 and < 0.2) and ≥ moderate MR (MR/LA ratio ≥ 0.2). Patients with any evidence of primary valve involvement, such as prior endocarditis, rheumatic valve disease, ruptured chordae or papillary muscle, congenital anomaly, or significant mitral annular calcification, were excluded. Patients with an ejection fraction < 50% were also excluded to preclude ventricular dysfunction-related FMR.

### 2.3 Voltage mapping and catheter ablation

After access to the LA with the Brockenbrough needle and a 8-French Swartz sheath (SRO, St. Jude Medical, MN), detailed endocardial voltage mapping of the LA was performed using a circular decapolar catheter (PV 12, APT) prior to ablation and in sinus rhythm in all cases. During the mapping procedure, patients in whom sinus rhythm could not be maintained were excluded from the study. Any area showing abnormal voltage was reassured with a 4-mm irrigated ablation catheter (Therapy Cool Path Duo or Cool Flex, St. Jude Medical, St. Paul, MN), in case of the presence of “false LVZs” resulting from inadequate contact between the circular catheter and LA wall.

To assess the LA substrate remodeling, LVZs were semi-quantitatively estimated and presented as low voltage index (LVI) as previously reported.^[10,11]^ The LA were divided into 6 separated zones, which were the roof between the left superior pulmonary vein (LSPV) and right superior pulmonary vein (RSPV), anterior wall between roof and MVA, posterior wall between roof and MVA, lateral wall between left atrial appendage (LAA) and MVA containing the ridge between LAA and LSPV, floor, and septum between right PV antrum and floor (Supplemental Figure 1). Each zone involving LVZs was rendered 1 point. We examined every beat to exclude mechanically induced premature beats. As elsewhere reported, LVZ was defined as sites of > 3 adjacent low-voltage points (bipolar voltage amplitude < 0.5 mV) and dense scar was defined as bipolar voltage amplitude < 0.1 mV.^[7]^

Intravenous heparin was administered after the transseptal puncture to maintain an activated clotting time of 300 to 350 seconds throughout the procedure. We used the 4-mm irrigated ablation catheter with power settings ≤ 40 W at the posterior wall and ≤ 50 W elsewhere to perform CPVI, an upper temperature limit of 43 °C, and a flow rate of 17 ml/min. Three-dimensional mapping navigation systems (EnSite Velocity, St. Paul, MN) were used to facilitate the ablation. Circumferential pulmonary vein isolation was performed by sequential application of radiofrequency energy at the antrum of the pulmonary veins. The end point was isolation of the pulmonary veins with proof of both exit and entrance block. A pace-and-ablate approach previously described was also employed to produce durable pulmonary vein isolation (PVI).^[12]^

### 2.4 Post-ablation management and follow-up

All patients were routinely treated with warfarin or dabigatran and previously ineffective antiarrhythmic medications for at least 3 months after the procedure, and were then stopped if no AF recurrence was found.

After the 3-month's blanking period, subsequent follow-up consisted of a clinical interview, electrocardiograms, and 24-hour Holter monitoring every 3 months for 1 year, and then every 6 months. In addition, the electrocardiograms were recorded at the time of any AF-related symptoms. Atrial fibrillation recurrence was defined as AF/atrial flutter/atrial tachycardia lasting >30 seconds recorded on the 12-lead electrocardiogram or Holter monitoring.^[13]^

### 2.5 Statistical analysis

The continuous variables were described as the mean ± standard deviation for normally distributed data and median (25%–75% quartile) for non-normally distributed data, and comparisons between groups were performed with Student's *t*-test (normally distributed data) or Mann–Whitney *U* test (non-normally distributed data). Categorical variables were described as counts and compared by χ^2^ analysis. Survival curves were generated with the Kaplan–Meier analysis and compared by log rank tests. Binominal logistic regression was used to calculate the odds ratio (OR) and 95% confidence interval (CI) for the presence of FMR or LVZs and Cox regression analysis was used to determine the independent predictors of AF recurrence, with a determination of hazard ratio (HR) and 95% CI for each variable in the model. The variables selected for testing in the multivariate analysis were those with *P* < 0.05 in the univariate models.

All tests were 2-tailed and a statistical significance was established at a *P* < 0.05. All analyses were performed using SPSS software version 18.0 (SPSS, Chicago, Illinois, USA).

## 3 Results

### 3.1 Baseline characteristics of the study population

A total of 132 patients, 100 men (75.8%), mean age 55.1 ± 9.6 years, with PAF were eligible for inclusion in the study. The median AF duration was 36 (18–84) months. Echocardiographic FMR was observed in 40 patients (29.6%), among whom 38 patients had mild FMR, while only 2 patients were detected with ≥ moderate FMR. Patient demographics are shown in Table 1. Multivariate analysis showed that older age, and larger LA diameter were associated with FMR (OR 1.054; 95% CI 1.002–1.109; *P* = 0.04; OR 1.130; 95% CI 1.006–1.269; *P* = 0.04, respectively) after adjustment for relevant clinical factors including sex, AF duration, and other comorbidities.

**Table 1:**
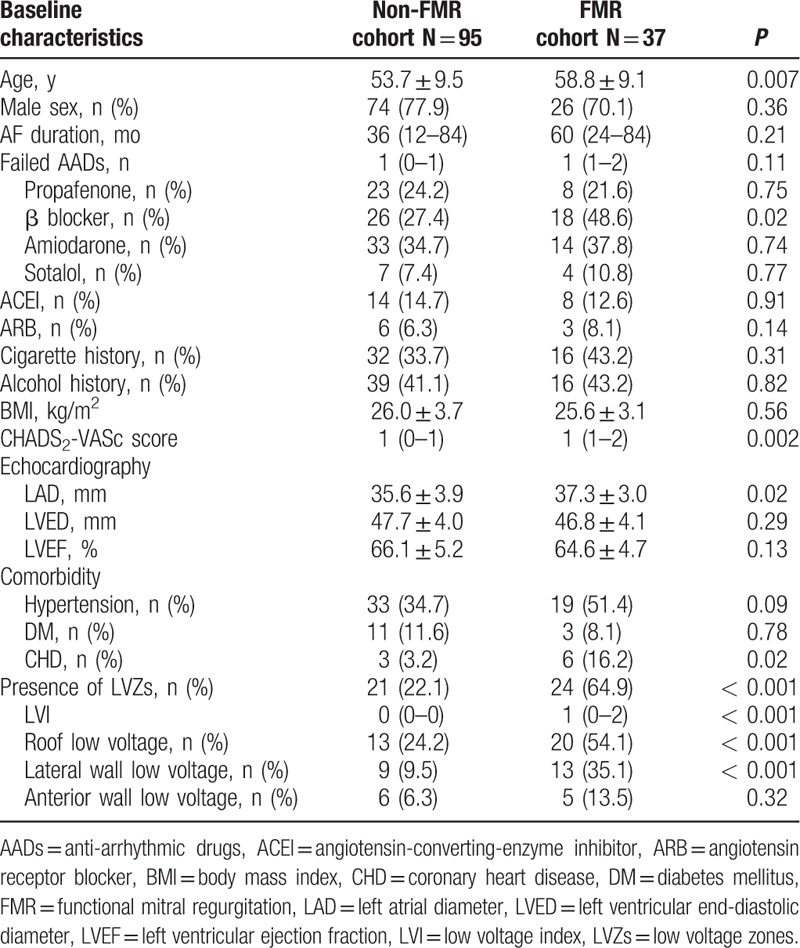
Baseline characteristics and voltage mapping parameters of the study population.

### 3.2 Functional mitral regurgitation and left atrium low voltage zones

**Figure 1:**
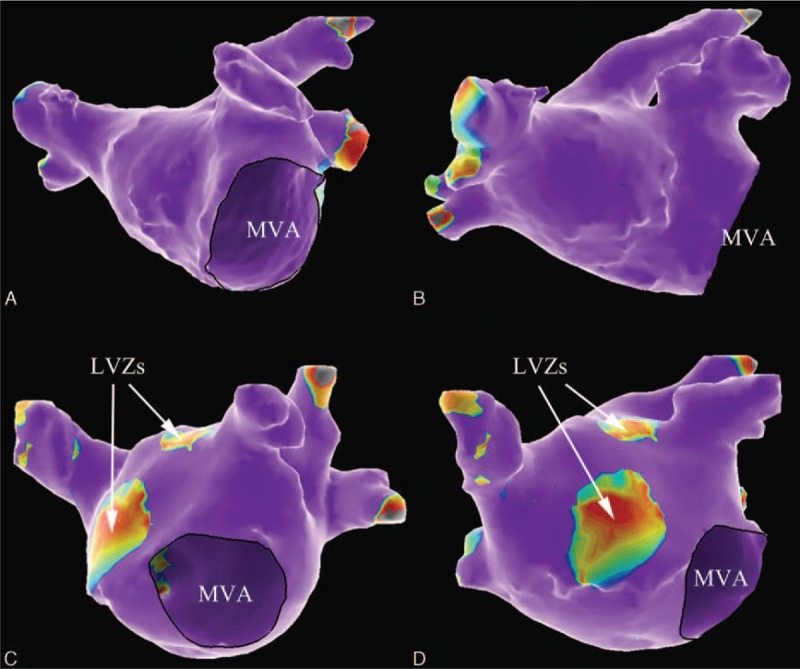
Examples of voltage mapping in patients with and without FMR. Voltage mapping in a patient without FMR (left atrium diameter 35 mm) in LAO (A) and RAO (B) view depicts a “healthy left atrium”, with no LVZ identified; while voltage mapping in a patient with FMR (left atrium diameter 42 mm) in LAO (C) and RAO (D) view shows 2 separate LVZs observed in the anterior wall and the roof (white arrows). FMR = functional mitral regurgitation, LAO = left anterior oblique, LVZs = low voltage zones, MVA = mitral valve annulus, RAO = right anterior oblique.

In total, LA LVZs in EAM were observed in 45 (34.1%) patients. There were 25 patients (18.9%) in whom only one division of LA was involved as LVZ, 15 patients (11.4%) whose LVI was 2, while 5 patients (3.8%) whose LVI was 3. The most frequently affected areas were the LA roof (33 patients; 25.0%) and lateral wall (22 patients; 16.7%), followed by the anterior wall (11 patients; 8.3%). Figure 1 shows the typical examples of EAM in patients with and without FMR.

**Figure 2:**
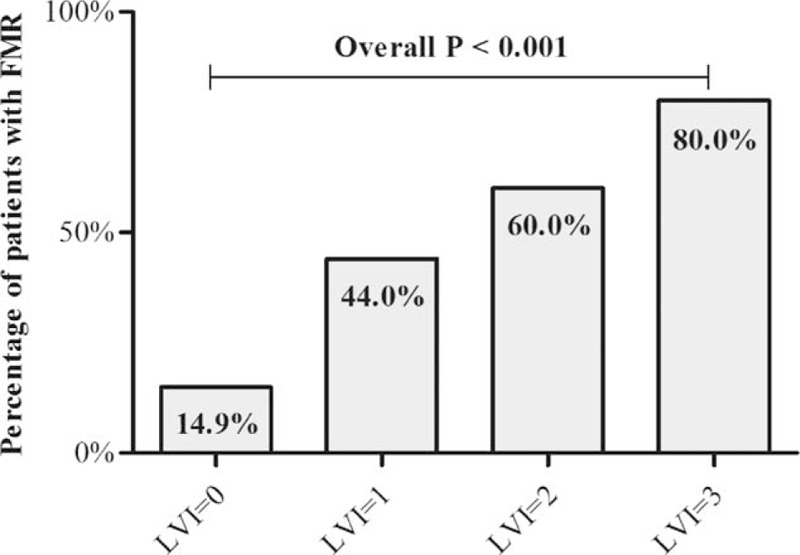
Percentage of patients with FMR in LVI of 0, 1, 2, and 3. A gradient distribution is observed with the overall *P* < 0.001. FMR = functional mitral regurgitation, LVI = low voltage index.

In FMR cohort, 24 patients (64.9%) were observed with LVZs while 21 patients (22.1%) were detected with LVZs in non-FMR group (*P* < 0.001). Moreover, the median LVI was significantly higher in patients with FMR (0 [0, 0] vs 1 [0, 2], *P* < 0.001) (Table 1). As is shown in Figure 2, from LVI of 0 to 3, the percentage of patients with FMR was 14.9%, 44.0%, 60.0%, and 80.0%, respectively (overall *P* < 0.001). In multivariate analysis, FMR was the strongest independent predictor of LVZs in LA: OR 7.286; 95% CI 3.023–17.562; *P* < 0.001. The probability of presence of LVZs in patients with FMR was 7 times as high as those without FMR. Additionally, AF duration also independently predicted the presence of LA LVZs (Table 2).

**Table 2:**
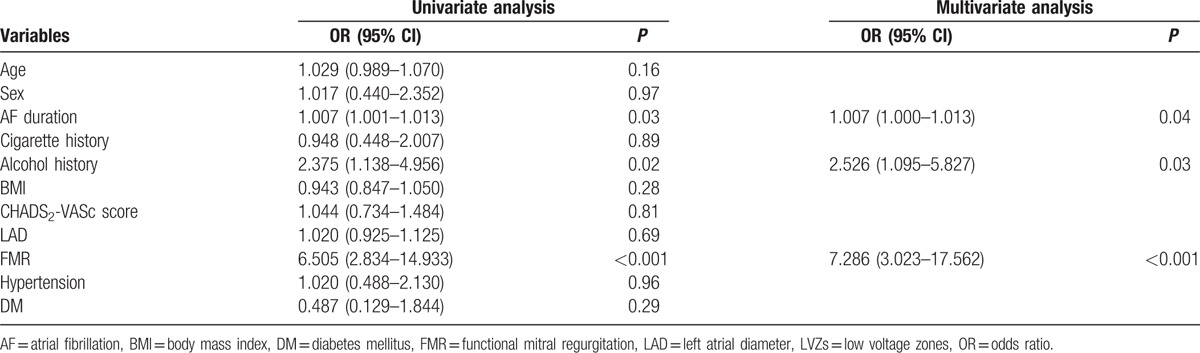
Univariate and multivariate analysis for the presence of LVZs.

### 3.3 FMR in relation to long-term outcomes after PAF ablation

During a mean follow-up of 22.9 ± 6.5 months (13–34 months), 10 patients were lost for follow-up. In the rest 122 patients, 38 patients (28.8%) experienced AF recurrence after a single CPVI procedure.

**Figure 3:**
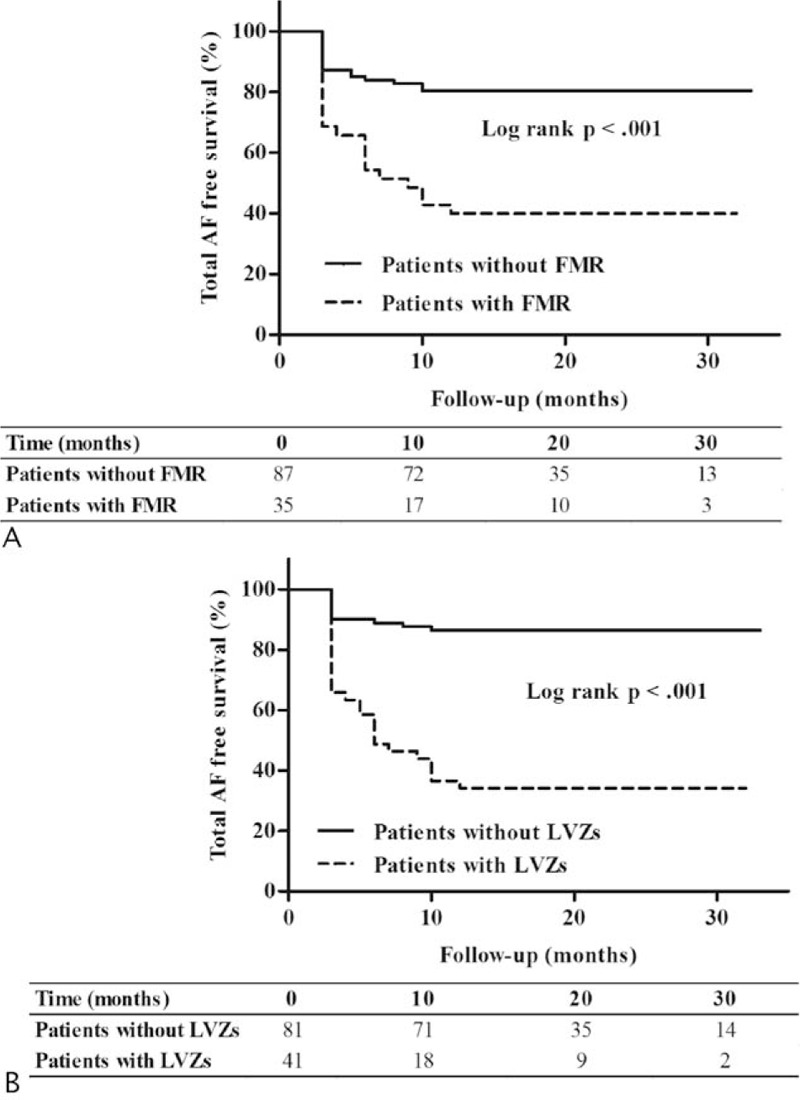
Kaplan–Meier curves for AF recurrence. (A) AF recurrence in FMR cohort and non-FMR cohort. (B) AF recurrence in patients without and with LVZ. AF = atrial fibrillation, FMR = functional mitral regurgitation, LVZ = low voltage zone.

In FMR cohort, 21 patients (60.0%) experienced AF recurrence, which was significantly higher than non-FMR cohort (17 patients, 19.5%, log rank *P* < 0.001) (Figure 3A). In addition, the AF recurrence rate was significantly higher in patients with LA LVZs (65.8% [27/41 patients] in LVZ group vs 13.6% [11/81 patients] in non-LVZ group, log rank *P* < 0.001) (Figure 3B).

Multivariate analysis showed that FMR independently predicted long-term outcomes post ablation in patients with PAF (HR 2.291, 95% CI 1.062–4.942, *P* = 0.03). The risk of AF recurrence in FMR cohort was more than twice as high as that in non-FMR cohort. Besides, LVI and alcohol intake history were also independent predictors of AF recurrence (HR 1.763, 95% CI 1.218–2.551, *P* = 0.003; HR 1.899, 95% CI 1.249–2.886, *P* = 0.003; respectively) (Table 3).

**Table 3:**
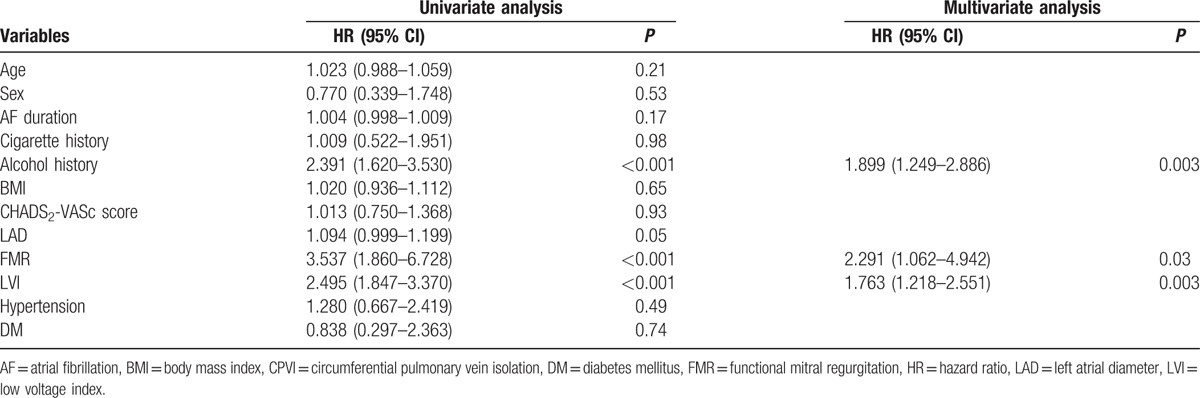
Univariate and multivariate analysis for AF recurrence after CPVI procedure.

## 4 Discussion

### 4.1 Main finding

The present study showed the following: FMR was found in 28% of patients with PAF, who were older and had larger LA diameter; FMR was strongly correlated with the presence as well as extent of LVZs; and FMR was associated with unfavorable ablation outcome after CPVI procedure. Therefore, FMR may serve as a predictor of advanced LA remodeling and further substrate modification may be needed to achieve a long-term success in this subgroup of patients. The present study is, to our knowledge, the first to prove the prognostic value and pathophysiologic significance of FMR.

### 4.2 FMR in patients with AF

Functional mitral regurgitation is most common in patients with left ventricular dysfunction and subsequent MVA dilation, typically in dilated and ischemic cardiomyopathy.^[14,15]^ Similarly, there is some evidence that FMR also exists in patients with AF and preserved left ventricular function, which is attributed to atrial remodeling and subsequent MVA dilation,^[1,2,4]^ although its prevalence varies among studies. Gertz et al^[1]^ found that 7.4% of the candidates for AF ablation have more than moderate FMR. In addition, FMR was the most common cause of MR among various etiologies.^[5]^ However, 29% of patients were identified with more than moderate FMR in a cohort of 480 individuals in the study by van Rosendael et al.^[2]^ In our study, 28% of the patients were detected with mild or even significant FMR. It is still controversial whether isolated MVA dilation resulting from AF could be sufficient to lead to significant FMR.^[1,4]^ Gertz et al^[1]^ revealed that LA remodeling and subsequent MVA dilation could result in significant FMR, which might be largely improved by sinus restoration. Of interest, in our study, only 2 patients (1.5%) were detected with moderate FMR, which was in accordance with Otsuji,^[4]^ who only observed modest FMR in 25 lone AF patients.

Various clinical factors were found to predict the presence of FMR. In a cohort of 770 patients, Gertz et al^[1]^ identified that patients with FMR were older, more likely to have persistent AF, and more frequently had hypertension. In our multivariate analysis, after adjustment for relevant clinical factors, older age and larger LA diameter were associated with FMR. All these characteristics were involved in DR-FLASH score, a novel scoring system which has been proposed and confirmed to predict LA remodeling in EAM.^[16]^ Therefore, FMR might serve as a potential marker for the substrate remodeling in AF patients.

### 4.3 FMR and LA substrate remodeling

It has long been recognized that the function of the LA plays a critical role for the well-functioning of mitral valve. Timely atrial contraction is important for appropriate mitral valve closing,^[17]^ and the strength and timing of atrial contraction may contribute to normal mitral valve function as well.^[18]^ Numerous studies have demonstrated that structural remodeling resulting from AF could lead to modest or even significant FMR, which is mediated by MVA dilation, although it remains controversial.^[1–4]^ Otsuji et al^[4]^ revealed that the MVA diameter of AF patients was significantly larger compared with healthy controls. Nevertheless, MVA dimensions were not related to FMR. In contrast, by echocardiography or computed tomography, Gertz^[1]^ and van Rosendael^[2]^ found that MVA dilation was independently associated with not only the presence but also the grade of FMR in AF patients with preserved left ventricular ejection fraction.

Electro-anatomical mapping has been an established tool for evaluating atrial substrate remodeling, which is mainly characterized by increased collagen deposition and matrix volume expansion.^[7,8]^ Combining the DE-MRI with EAM, Oakes et al^[7]^ elegantly demonstrated the close relationship between LVZ and fibrotic tissue. In the present study, we found that FMR was strongly associated with the presence and extent of LVZs. The probability of presence of LVZs in patients with FMR was 6 times higher than those without FMR. Moreover, the semi-quantitatively defined LVI was significantly higher in patients with FMR. Therefore, we demonstrate the close link between FMR and substrate remodeling from the point of view of electrophysiology, which is in accordance with observation from previous echocardiographic studies.

### 4.4 FMR and ablation outcome post CPVI

Various clinical factors have been proposed as indicators for AF recurrence post ablation, including LA enlargement, LA scarring, and elevated troponin level post ablation.^[19–21]^ Among them, MR was demonstrated to have a detrimental impact on AF ablation outcomes.^[5]^ Moreover, the grade of MR severity had positive correlation with the recurrence rate.^[6]^ However, all these studies included patients both with and without primary valvular abnormalities, which hardly specialized to patients with FMR.^[5]^ In the present study, we exclusively enrolled patients without a history of mitral valve disease and observed that FMR independently predicted the long-term AF recurrence post ablation. In patients with FMR, the risk of AF recurrence was more than twice as high as those without FMR. We believe the most plausible explanation for this finding might be the adverse atrial substrate remodeling. Indeed, as mentioned above, FMR was associated strongly with the presence and extent of LVZs in EAM, which indicated the predictive value of FMR on atrial remodeling.

Although focal triggering originated from pulmonary vein was believed to be the predominant mechanism for PAF,^[22]^ substrate remodeling was also demonstrated to be present in certain number of patients and associated with poor ablation outcome.^[10,23]^ Recently, Rolf et al^[23]^ proposed an individualized ablation strategy based on the presence and location of LVZs. In accordance with our observation, patients with LVZs undergoing pure PVI had a higher recurrence rate, irrespective of AF type. By adding further lesions across the LVZs, they could improve the ablation outcome to a level comparable with those without LVZ undergoing pure PVI.^[23]^ Likewise, we hypothesize that exclusive CPVI may not be sufficient to maintain sinus rhythm in the long term and further substrate modification may have to be considered in these patients, which, however, merits further investigation.

## 5 Limitation

Firstly, the present study is a retrospective cohort study. Secondly, we could hardly ascertain that all the patients have Carpentier I MR because the accurate Carpentier types of MR in our study population were not available. Nevertheless, we excluded common cause of primary MR including rheumatic heart disease and severe annulus calcification. Thus, we believe that there were very few, if any, patients with primary MR included in the study. Thirdly, all patients underwent 24-hour-Holter monitoring, rather than implanted loop recorder during follow-up, which may potentially underestimate the recurrence rates. Finally, in this study, we could only prove the association between FMR and LA remodeling rather than causal relationship. Therefore, further studies are still needed.

## 6 Conclusions

Functional mitral regurgitation is a common finding in candidates for PAF ablation, which is strongly correlated with the presence as well as extent of LVZs in EAM. Furthermore, FMR is associated with poor ablation outcome. Therefore, patients with FMR tend to have more advanced substrate remodeling, which facilitate the AF recurrence with pure CPVI. However, since our study is retrospective and therefore only hypothesis-generating, the question of whether patients with AF and FMR could benefit from additional substrate modification should be addressed in a prospective randomized controlled study.

## Supplementary Material

Supplemental Digital Content
