# Supplementary material for: Functional mitral regurgitation: predictor for atrial substrate remodeling and poor ablation outcome in paroxysmal atrial fibrillation
Source: Medicine (Baltimore). 2016 Jul 29;95(30):e4333. doi: 10.1097/MD.0000000000004333 (PMC5265852; doi:10.1097/MD.0000000000004333)

**Supplemental figure legend**

**Supplemental figure 1** Segmentation of left atrium (LA) in AP (A), PA (B), LAO (C), RAO (D) view. Numbers 1-6 indicate anterior wall, roof, lateral wall, posterior wall, septum, and floor respectively. LSPV, left superior pulmonary vein; LIPV, left inferior pulmonary vein; RSPV, right superior pulmonary vein; RIPV, right inferior pulmonary vein; LA, left atrium, LAA, left atrial appendage; MVA, mitral valve annulus, AP, anteroposterior, PA, posteroanterior, LAO, left anterior oblique, RAO, right anterior oblique.


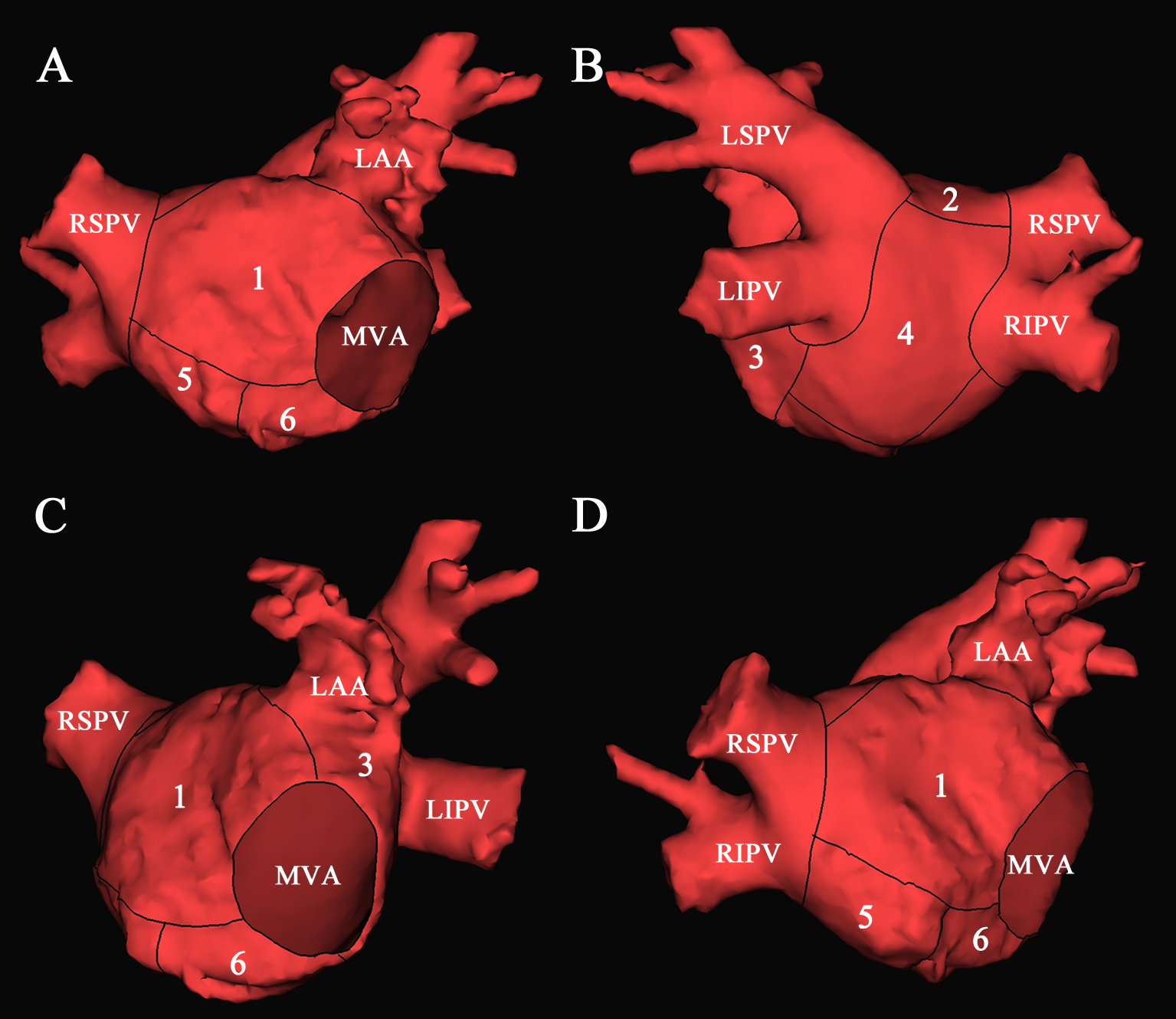

Supplement: Supplemental Digital Content [file medi-95-e4333-s001.doc]
